# A Highly Compact and Isolated Triple-Band MIMO Antenna for Wireless Capsule Endoscopy and Cardiac Implant

**DOI:** 10.3390/mi17030296

**Published:** 2026-02-27

**Authors:** Tahir Bashir, Guanjie Feng, Shunbiao Chen, Yunqi Cao, Wei Li

**Affiliations:** 1 College of Integrated Circuit Science and Engineering, Nanjing University of Posts and Telecommunications, Nanjing 210023, China; tahir.bashir@njupt.edu.cn (T.B.); b22030509@njupt.edu.cn (G.F.); b22022219@njupt.edu.cn (S.C.); 2 College of Control Science and Engineering, Zhejiang University, Hangzhou 310027, China; caoyunqi@zju.edu.cn; 3 School of Mechanical Science and Engineering, Huazhong University of Science and Technology, Wuhan 430074, China

**Keywords:** implantable antenna, triple-band, MIMO, capsule endoscopy, leadless pacemaker, coupling

## Abstract

This work presents a highly compact triple-band multi-input-multi-output (MIMO) implantable antenna for wireless capsule endoscopy (WCE) and leadless cardiac pacemakers. The proposed antenna operates at industrial, scientific, and medical (ISM) bands of 2.400 to 2.480 GHz and 5.725 to 5.875 GHz for data telemetry and the wireless medical telemetry service (WMTS) band of 1.395 to 1.432 GHz for efficient wireless power transfer. The four-element design measures 8.5 × 8.5 × 0.26 mm^3^ and achieves low mutual coupling through a planar four-port configuration with optimized inter-element spacing. The antenna is integrated within realistic capsule devices containing batteries, sensors, and electronic components, and evaluated in both homogeneous and realistic heterogeneous body phantoms, including the large intestine and heart. The design yields maximum reflection coefficients of −26.15 dB, −15 dB, and −36.32 dB, −10 dB bandwidths of 260 MHz, 160 MHz, and 160 MHz, mutual coupling of −37.74 dB, −44.55 dB, −26.48 dB, and peak realized gains of −35 dBi, −25 dBi, and −15 dBi at 1.4 GHz, 2.45 GHz, and 5.8 GHz, respectively. Specific absorption rate (SAR) analysis satisfies implantation safety limits. Link budget analysis confirms reliable communication over distances > 20 m in all bands with data rates up to 100 Mbps. MIMO channel parameters such as envelope correlation coefficient (ECC) and diversity gain (DG) remain within acceptable limits. Owing to its multi-band operation, miniaturization, and isolation, the proposed four-port antenna is a good candidate for next-generation WCE and leadless pacemaker systems.

## 1. Introduction

In recent years, biomedical engineering has experienced substantial advancements, driving the development of implantable biomedical devices (IMBDs) with enhanced diagnostic, therapeutic, and monitoring capabilities [1]. These systems rely on wireless communication for targeted treatment and real-time physiological data acquisition, making implantable antennas essential components [2]. However, antenna design for IMBDs remains challenging due to high requirements on miniaturization, impedance matching, high-speed data transmission, multi-band, and patient safety [3]. With the increasing demand for compact, multifunctional IMBDs, there is an urgent need for miniaturized multiband antennas that can operate efficiently within limited device space and lossy biological tissues [4]. While SISO implantable antennas have been widely investigated [5,6,7,8], they are restricted by low spectral efficiency and limited data throughput. MIMO implantable antennas have therefore been introduced to enhance link reliability, data rates, and multi-band operation [9,10,11,12]. Despite integrating multiple antenna elements, several MIMO designs remain single-band, occupy relatively large volumes, or are designed to satisfy the multi-frequency and miniaturization needs of modern IMBDs. Considerable efforts have focused on achieving multiband operation, high data rates, and miniaturization in implantable antennas [13,14,15,16,17,18,19,20,21,22]. For instance, a two-element implantable MIMO antenna operating at 402 MHz was presented in [13]; however, its relatively large dimensions of 22 × 22 × 0.635 mm^3^ limit its suitability for modern IMBDs. In [14], a four-element MIMO antenna incorporating an electromagnetic band-gap structure was proposed, yet it suffers from inadequate isolation. In [15], the work presents a compact (5.12 × 5.12 × 4.6 mm^3^) dual-band cubic MIMO antenna that leverages radiation diversity and self-isolation to ensure reliable high-speed data transmission. However, a notable shortcoming of this work is the structural rigidity of the cubic geometry, which may limit its integration into emerging flexible or soft-bodied biomedical implants that require conformal movement within the human body. In [16], a dual antenna system for implantable biotelemetry has been designed to transmit real-time physiological data from inside the human body to an external receiver. However, a proposed antenna system’s relatively large dimensions limit its suitability for compact biomedical devices. In [17], a quadrilateral cubic MIMO system has been proposed that leverages 3D spatial diversity to maintain robust, orientation-insensitive links with good isolation. However, the cubic structure presents a significant disadvantage due to its increased volume and lack of conformability compared to flexible or compact designs, posing challenges for implantation in spatially constrained anatomical regions. To achieve further miniaturization, researchers have also explored conformal antenna configurations that utilize the inner and outer surfaces of capsule-type devices [20,21,22]. Although these configurations reduce size, their overall dimensions typically confine them to capsule-based platforms and limit applicability in IMBDs with spatial constraints. Overall, existing implantable antennas still face limitations such as large physical size, restricted frequency operation, low isolation, highly directional radiation, or device-specific compatibility. This highlights the need for an implantable antenna offering a small footprint, multiband functionality, high isolation, and applicability across diverse IMBD systems.

**Figure 1:**
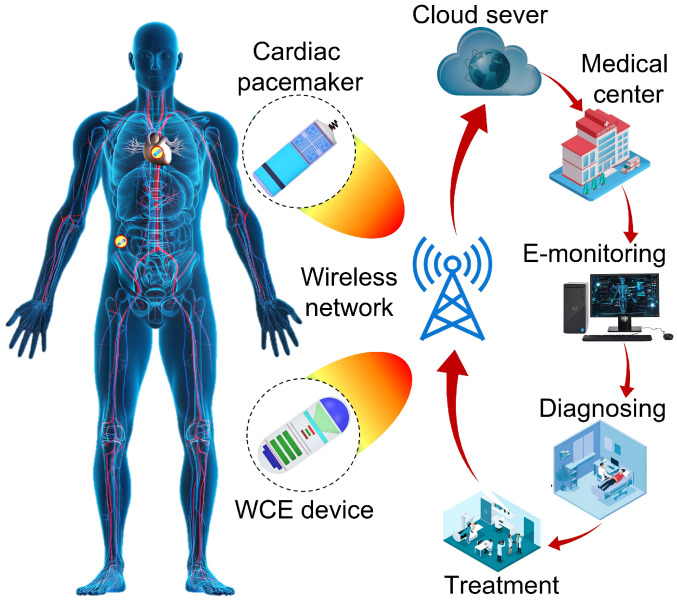
An overview of the proposed compact triple-band MIMO antenna designed for integration into biotelemetric systems.

Designing a multiband, compact MIMO antenna for modern IMBDs while simultaneously ensuring high isolation, enhanced channel capacity, and an omnidirectional-like radiation pattern remains a significant challenge, particularly given the size constraints of diverse IMBDs. The proposed work addresses these challenges through the development of a highly efficient, isolated, miniaturized triple-band MIMO antenna suitable for multiple wireless biomedical implant platforms. The key contribution of this study lies in the design and demonstration of a highly compact four-port implantable antenna optimized for gastrointestinal (GI) tract capsule endoscopy and cardiac leadless pacemaker systems. Figure 1 presents a conceptual overview of the proposed wireless patient monitoring system for WCE and cardiac applications. In this system, a triple-band antenna-integrated implantable device transmits signals at 2.45 GHz to an external receiver, which subsequently forwards the data via the Internet (cloud) to a medical facility. The doctor can monitor the patient in real time, make diagnostic assessments, and generate treatment plans. To support high data rates, miniaturization, and multi-protocol communication, the antenna operates in the WMTS 1.4 GHz, ISM 2.45 GHz, and 5.8 GHz bands, enabling both wireless power transfer and data telemetry. The four-element MIMO structure occupies a volume of only 18.785 mm^3^ and achieves high isolation without external decoupling components. It exhibits reflection coefficients of −26.15 dB, −15 dB, and −36.32, −10 dB bandwidths of 260 MHz, 160 MHz, and 160 MHz, isolation levels of −37.74 dB, −44.55 dB, and −26.48, and realized gains of −35 dBi, −25 dBi and −15 dBi in the WMTS and ISM bands, respectively. To demonstrate the applicability of the proposed antenna for various biomedical applications, it was encapsulated within IMBDs and evaluated in both homogeneous and heterogeneous tissue models. The safety of the proposed MIMO antenna was analyzed by calculating the SAR to ensure compliance with human-body exposure limits. Additionally, link-budget analysis was performed to evaluate the reliability of wireless communication between the implantable MIMO antenna and external devices, confirming real-time data transmission over several meters at high data rates. Furthermore, key MIMO channel parameters, including ECC and DG, were analyzed, demonstrating that the proposed design meets the requirements for robust MIMO operation. Table 1 compares the proposed MIMO antenna with reported implantable MIMO designs, highlighting its key advantages for biomedical applications.

## 2. Capsule-like Implantable Devices and SISO Performance

**Figure 2:**
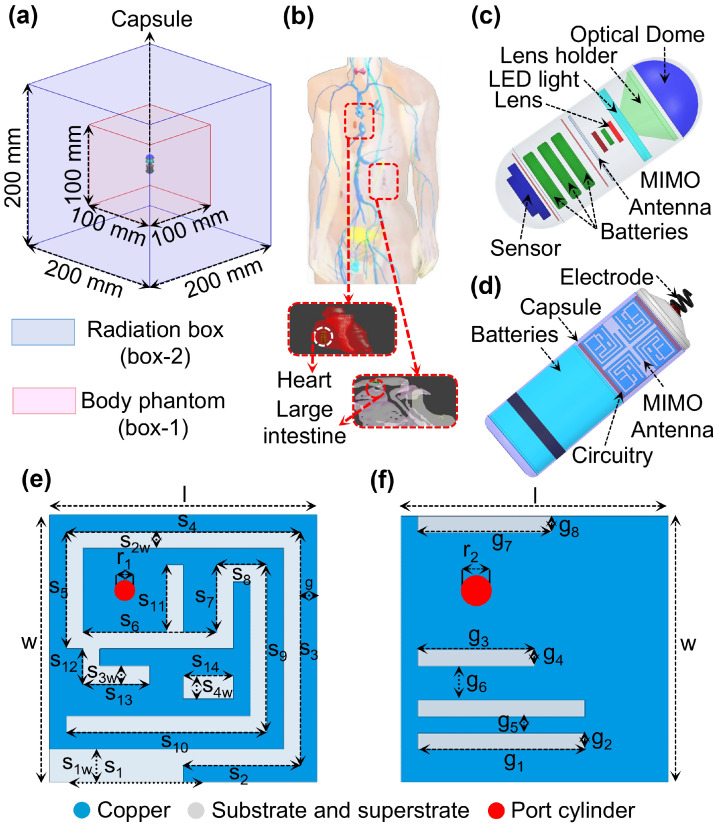
(**a**) Homogeneous setup (**b**) heterogeneous (**c**) WCE (**d**) cardiac pacemaker (**e**) patch (**f**) ground (l = w = 4, S_1_ = 1.77, S_1w_ = 0.5, S_2_ = 1.52, S_3_ = S_4_ = 3.5, S_5_ = 1.75, S_6_ = 2.5, S_7_ = S_11_ = 1.25, S_8_ = 0.75, S_9_ = 2.5, S_10_ = 3, S_12_ = 0.78, S_13_ = 1, S_14_ = 0.75, g = 0.25, S_2w_ = S_3w_ = 0.25, S_4w_ = 0.35, g_1_ = g_5_ = 2.5, g_3_ = 1.75, g_7_ = 2, g_2_ = g_4_ = g_5_ = g = 0.25, g_6_ = 0.5, r_1_ = 0.125, r_2_ = 0.2).

**Figure 3:**
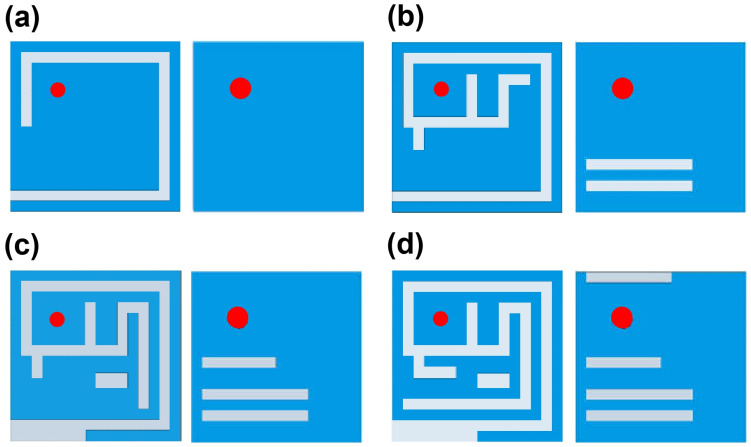
Design evolution of triple-band Ant-I (**a**) step I (**b**) step II (**c**) step III (**d**) step IV.

**Figure 4:**
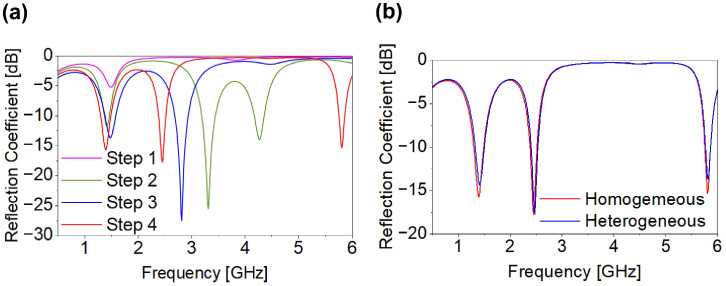
(**a**) Performance evolution of Ant. I (**b**) |S_11_| of Ant-1.

Biocompatible coatings are commonly employed in implantable antennas to provide sufficient insulation from surrounding biological tissues. However, for practical implementation and comprehensive system-level evaluation, the antennas must be embedded within fully encapsulated implantable devices. In this study, a realistic implantable capsule device was first modeled, incorporating key device components such as battery, sensors, and electronic circuitry. The antenna was then integrated within this capsule, and all geometrical modifications and optimizations were performed in the presence of these components. Therefore, the reported antenna characteristics, including reflection coefficient, mutual coupling, radiation behavior, and MIMO performance, inherently account for the electromagnetic influence of the device components. This integrated design approach reflects practical implantable scenarios, where the antenna must operate within a fully encapsulated device prior to implantation inside the human body. The device layouts considered in this paper are shown in Figure 2. The air-filled WCE measures 24.65 mm in length with a 5.5 mm radius, comprising two lids of length 5.5 mm and a 13.65 mm cylindrical section, as shown in Figure 2c. The air-filled leadless pacemaker is 24.5 mm long with an 11 mm diameter and a 1.2 mm upper lid, as shown in Figure 2d. Both devices have 0.25 mm-thick walls and are designed using alumina (Al_2_O_3_) with ε_r_ = 9.8 and tanδ = 0.006. For simulation, electronic circuitry is modeled as a substrate layer between two PEC plates, using Rogers RT/duroid 6010 (ε_r_ = 10.2, tanδ = 0.0023, 0.4 mm thick). The antenna is designed using HFSS and Sim4Life in a dedicated in-body simulation environment. To realize realistic conditions, it is centered in a homogeneous large-intestine phantom with frequency-dependent dielectric properties whose relative permittivity is 56.1, 53.9, 48.5 and electrical conductivity is 1.33, 2.03, 5.57 S/m at 1.4, 2.45, and 5.8 GHz, respectively. The phantom (Box-1) has dimensions 100 × 100 × 100 mm^3^ and is enclosed by a 200 × 200 × 200 mm^3^ radiation boundary (Box-2), with an implantation depth of 50 mm, as shown in Figure 2a. Heterogeneous-tissue validation is performed in Sim4Life using the same depth as shown in Figure 2b. The MIMO design begins with a SISO antenna (Ant. I), as shown in Figure 2e,f, measuring 4.0 × 4.0 × 0.26 mm^3^. It includes a superstrate, substrate, radiating patch, and a partially slotted ground. Miniaturization is achieved by employing a meandered structure, high-permittivity Rogers RT/duroid 6010 (ε_r_ = 10.2, tanδ = 0.0023) and a thin 0.13 mm substrate. To further improve the isolation between antenna and circuitry within the capsule devices, a superstrate identical in size and material to the substrate is incorporated above the radiating patch. The antenna is fed through a 50 Ω coaxial port of 0.4 mm diameter. The Ant-I structure achieves triple-band and the desired |S_11_| through a four-step optimization process. Figure 3 shows the design evolution of the triple-band Ant-I MIMO system. The initial design used a square patch with a rectangular slot (s_1_ − s_5_) and a full ground plane, resonating at 1.50 GHz with |S_11_| = −5.20 dB as shown in Figure 4a. In step II, additional patch and ground slots (s_6_, s_7_, s_8_, s_11_, s_12_, and g_1_) generated multiple resonances at 1.41, 3.12, and 4.26 GHz with |S_11_| of −11.64, −25.51, and −13.97 dB. Step III introduced s_9_, s_14_, s_1w_, and g_3_, shifting the resonances to 1.48 and 2.81 GHz with |S_11_| of −13.61 and −27.48 dB. Final tuning in step IV added slots s_10_, s_13_, s_14_, and g_7_, enabling accurate triple-band operation at 1.4, 2.45 GHz and 5.8 GHz with |S_11_| = −16 dB, −18 dB, and 15.5 dB as shown in Figure 4a. The antenna maintains linear polarization throughout the design evolution process. Figure 4b shows the reflection coefficient performance of Ant. I in both environments with good agreement with each other.

## 3. Two and Four-Port MIMO Antenna and Performance

**Figure 5:**
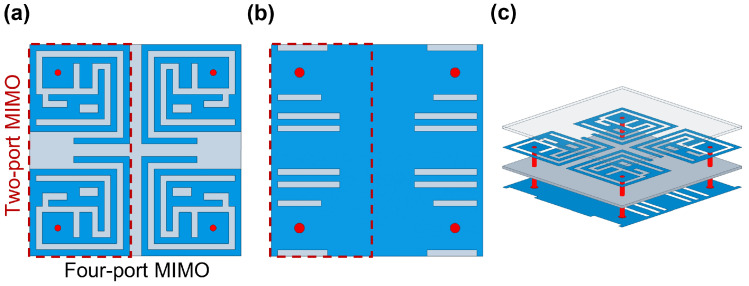
(**a**) Top view of MIMO antenna, (**b**) bottom view, (**c**) exploded 3D view.

**Figure 6:**
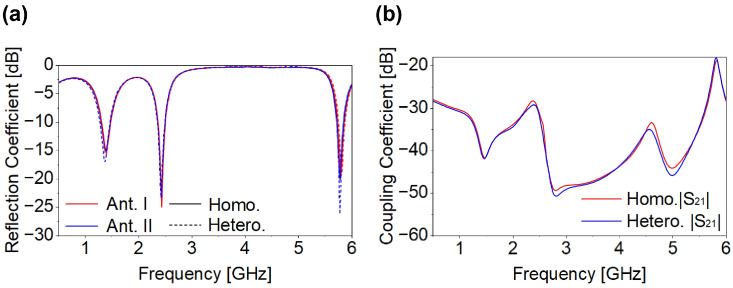
Two-port MIMO (**a**) reflection and (**b**) coupling coefficient.

**Figure 7:**
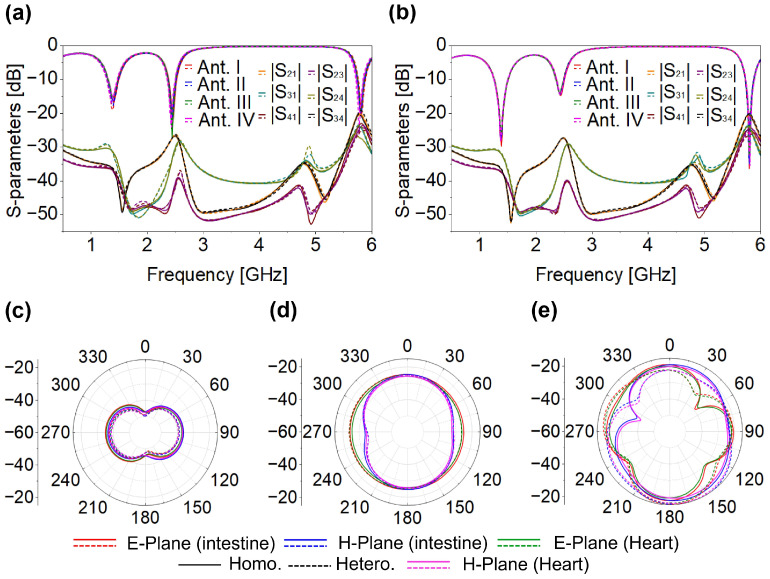
Four-port MIMO (**a**) WCE (**b**) pacemaker (**c**) radiation pattern at 1.4 GHz (**d**) radiation pattern at 2.45 GHz (**e**) radiation pattern at 5.8 GHz.

After a detailed parametric investigation, the finalized Ant-I element was integrated into the MIMO configuration. Two identical SISO antennas were arranged in opposite orientations, as shown in Figure 5. Designing an implantable MIMO system is challenging because multiple closely spaced elements must be tuned similarly with minimum mutual coupling. Although the SISO design provides a basis, its characteristics do not directly contribute to the MIMO system due to strong inter-element coupling. Thus, additional coupling-reduction techniques were applied to maintain the performance without changing the original Ant-I geometry, including its patch and ground plane. As shown in Figure 6, the two-port MIMO antenna maintained the intended resonances without detuning and achieved the desired reflection and coupling coefficient characteristics. Mutual coupling remains below −39.19 dB, −29.34 dB, and −20 dB at 1.4 GHz, 2.45 GHz, and 5.8 GHz, respectively. Based on the two-port MIMO design and its good reflection and coupling coefficient performance, a four-port MIMO configuration was subsequently developed. The proposed 4 × 4 MIMO antenna is formed by employing the same optimized single antenna (Ant-I) element and arranging them with appropriate inter-element spacing. Accordingly, the reflection coefficients (|S_11_|, |S_22_|, |S_33_|, and |S_44_|) and mutual coupling parameters are presented for all antenna elements to demonstrate consistent S-parameters performance. Since all antenna elements have identical geometry and loading conditions, their radiation characteristics are inherently the same; therefore, the radiation pattern of a single antenna element is shown as a representative result. The proposed four-port MIMO systems occupy compact dimensions of 8.5 × 8.5 × 0.26 mm^3^, corresponding to a total volume of 18.785 mm^3^. The antenna footprint was selected to ensure compatibility with capsule-type implants; as discussed in Section 2, the proposed structure meets the spatial constraints of both WCE and cardiac leadless pacemaker devices, enabling seamless integration as illustrated in Figure 2. To validate its applicability for WCE and cardiac pacemakers, the four-port MIMO antenna was evaluated within both the large intestine and the heart phantoms. Figure 7a,b shows the reflection and coupling coefficient under homogeneous and heterogeneous tissue conditions. For the intestine, reflection coefficients range from −15.24 dB to −18.27 dB at 1.4 GHz, from −18.76 dB to −27.09 dB at 2.45 GHz, and from −18.03 dB to −20.26 dB at 5.8 GHz, as shown in Figure 7a. In the heart phantom, values vary from −23.19 dB to −26.15 dB at 1.4 GHz, −14.56 dB to −15.74 dB at 2.45 GHz, and −30.72 dB to −36.32 dB at 5.8 GHz, as shown in Figure 7b. The proposed MIMO antenna maintains stable resonance within the target frequency bands and achieves the desired reflection characteristics across all simulated environments. The coupling coefficients |S_21_|, |S_31_|, |S_41_|, |S_23_|, |S_24_|, and |S_34_| remain below −31.56 dB, −27.78 dB, and −20.10 dB for both phantoms at respective frequency bands as shown in Figure 7a,b. Due to symmetry (|S_mn_| = |S_nm_|), the remaining coefficients are omitted. Although antenna performance varies with changes in the implantation medium, the proposed four-port MIMO antenna consistently maintains its intended resonant behavior, impedance and isolation characteristics. The far-field characteristics in both simulation environments exhibit good agreement, as shown in Figure 7c–e. The simulated peak gains are −35 dBi for the intestine and −35 dBi for the heart at 1.4 GHz, −23.8 dBi and −25 dBi at 2.45 GHz, −15 dBi and −15 dBi at 5.8 GHz. Lower gains at 1.4 GHz arise from increased dielectric loss in biological tissues at lower frequencies.

## 4. Decoupling and Detuning Analysis of MIMO Antenna

High coupling in MIMO antenna systems can significantly degrade overall system performance, particularly in terms of signal isolation, diversity gain, and spatial diversity. This issue is especially critical in biomedical applications, where maintaining reliable transmission quality is challenging due to the lossy nature of human body tissues. Various decoupling techniques have been proposed to mitigate coupling in MIMO systems, including stubs, strips, neutralization lines, defected ground structures, electromagnetic bandgap (EBG) structures, and metamaterial structures. However, each of these techniques has inherent limitations. A recent study [23] demonstrated the use of an inductor between two antennas for biomedical applications, achieving coupling levels of −35 dB and −31 dB at two resonant frequencies. Similarly, another study [24] reported isolation levels of −35 dB by integrating inductors between antenna elements in implantable systems. While incorporating inductors is an effective approach to reduce coupling, its implementation presents significant challenges, increases fabrication complexity and integration difficulties. These limitations highlight the need for simple alternative approaches in achieving effective decoupling.

**Figure 8:**
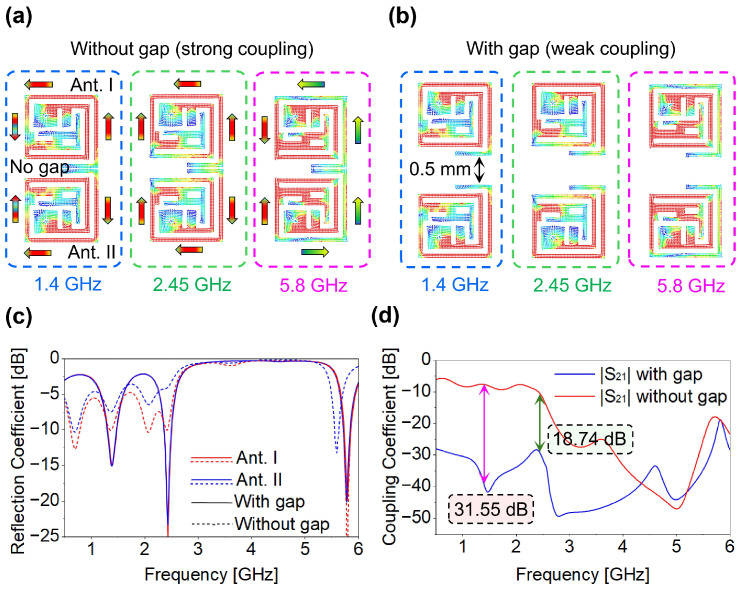
Frequency detuning and decoupling analysis of two-port MIMO antenna with and without inter-element spacing (**a**) current distribution without gap (**b**) with gap (**c**) reflection coefficient (**d**) coupling coefficient.

In this study, we achieved a significant reduction in coupling without introducing any modifications to the antenna structure after MIMO configuration generation, without employing external components. At all operating frequencies, the current distribution along the lower sides of the antennas is relatively weak compared to the other side, as depicted in Figure 8. Additionally, the current flows in the same direction across the connected sides in two- and four-port MIMO systems, generating strong currents and enhancing coupling between them at both frequencies. To address the adverse effects of high coupling, an inter-element spacing of about 0.5 mm was introduced between closely connected antenna elements, as shown in Figure 8b. By inserting a space between them, the reduction in coupling strength at resonant frequencies can be reduced. Due to high coupling, the designed MIMO experienced a strong frequency detuning effect at operating frequencies, as shown in Figure 8c. By carefully selecting the optimal spacing between them, we not only mitigated the coupling effect but also maintained the same frequency tuning as achieved in the single antenna design, as illustrated in Figure 4b. The results clearly demonstrate that inter-element spacing led to a substantial reduction in coupling, with |S_21_| decreasing 31.55 dB, improving from −7.64 dB to −39.19 dB at 1.4 GHz, while at 2.45 GHz |S_21_| decreasing 18.74 dB, improving from −10.7 dB to −29.34 dB, as depicted in Figure 8d. In prior works [23,24], 2 × 2 antenna configurations with inter-element gaps of 0.3 mm and 0.4 mm were used, and inductors were integrated to reduce coupling. In contrast, the proposed 4 × 4 MIMO antenna uses a slightly larger inter-element spacing of 0.5 mm, achieving high isolation without additional components. Despite having more elements, the overall antenna size remains smaller than the referenced 2 × 2 designs [23] due to optimized geometry and spacing. This approach balances the tradeoff between element spacing, mutual coupling, and compactness, providing a simple, effective and practical solution for implantable devices. To illustrate the impact of inter-element spacing on isolation performance, we have presented the two-port MIMO antenna case demonstrating the isolation improvement. Notably, isolation improvements in 4-port MIMO have been achieved at the corresponding resonant frequencies, as depicted in Figure 7a,b. Designing a highly compact and small-sized antenna with a MIMO configuration while ensuring high isolation presents significant challenges. Despite this, the proposed MIMO antenna achieves isolation levels of −39.19 dB and −29.34 dB, well beyond the typical threshold of −15 dB. This demonstrates its effectiveness in multi-tissue applications while maintaining a compact profile.

## 5. SAR Evaluation for Safe Implantation

**Figure 9:**
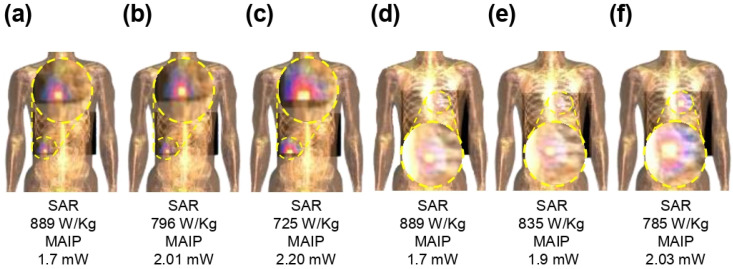
Normalized1-g SAR distribution and MAIP of the proposed MIMO antenna (**a**–**c**) intestine at 1.4 GHz, 2.45 GHz, and 5.8 GHz (**d**–**f**) heart at 1.4 GHz, 2.45 GHz, and 5.8 GHz.

To ensure patient safety, it is essential to calculate the electromagnetic exposure associated with the proposed implantable MIMO antenna. The SAR works as a key indicator for evaluating the biological effects of EM fields on human tissues. According to the IEEE C95.1–2019 standards, the maximum permissible SAR is 1.6 W/kg for 1 g tissue and 2 W/kg for 10 g tissue samples [25]. Various simulation platforms have been employed for SAR assessment, including CST with the Gustav voxel model, Remcoms 3D voxel models, and Sim4Life. Owing to its high-fidelity anatomical representations, in this work, SAR evaluation was conducted using Sim4Life with the MIMO antenna positioned within heterogeneous heart and large intestine structures of realistic human phantoms, as illustrated in Figure 2b. Each antenna element was excited with 1 W input power. The resulting SAR values, corresponding maximum allowable input powers, and distribution at all resonant frequencies are shown in Figure 9. Considering the power requirements for implantable biomedical systems (−16 dBm), the proposed MIMO antenna remains well within safe limits across respective operational frequency bands.

## 6. Communication Analysis of the Proposed MIMO Antenna

The wireless telemetry performance of the proposed antenna was evaluated through a detailed transmission analysis. For the uplink, the implantable antenna operates as the transmitting T_x_ element. The link model is expressed by the following equations:

(1) $$LM=P_{a}-P_{r}$$

where LM denotes the link margin, P_a_ is the available power, and P_r_ is the received power. The available power is computed as:

(2) $$P_{a}=P_{T}+G_{T}+G_{R}-L_{p}-PL$$

where PL, is the path loss, can be expressed as follows:

(3) $$PL\left(dB\right)=10\gamma {log}_{10}\left(\frac{d}{d_{0}}\right)+20{log}_{10}\left(\frac{4\pi d_{0}}{\lambda_{0}}\right)+X\sigma$$

**Figure 10:**
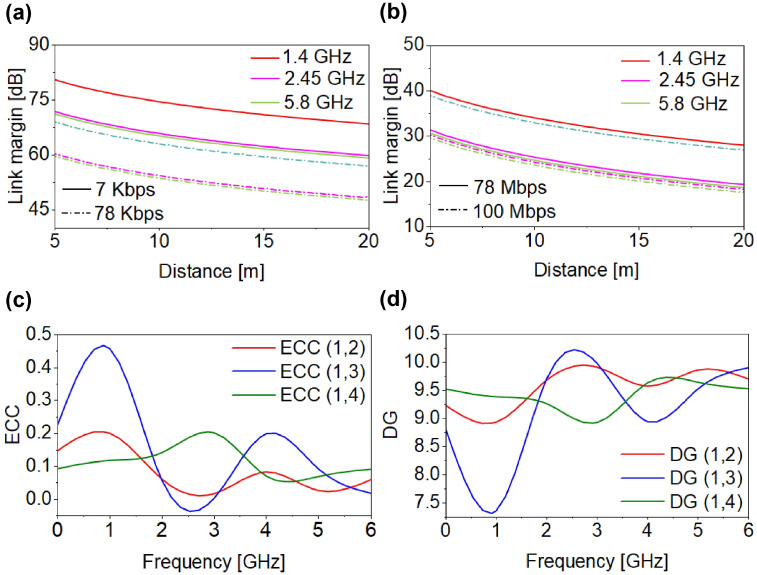
(**a**) Link margin analysis at Kbps (**b**) Mbps (**c**) ECC (**d**) DG.

In this expression, γ is the path-loss exponent, d_0_ is the reference distance (d_0_ < d), d is the separation between the T_x_ and R_x_ antennas, λ_0_ is the free-space wavelength, and X_σ_ represents the shadowing term. As illustrated in Figure 10a,b, for data rates of 7 Kbps, 100 Kbps, 78 Mbps, and 100 Mbps at 1.4 GHz, 2.45 GHz, and 5.8 GHz, the proposed MIMO antenna achieves a reliable high-speed (100 Mbps) link over distances exceeding 20 m at all frequencies.

## 7. ECC and DG Performance Analysis

The performance analysis of a MIMO antenna system, which incorporates multiple radiating elements, requires a more comprehensive evaluation than that of single-antenna designs. In particular, inter-element correlation must be examined to determine the system’s suitability for practical use. Key parameters, including ECC and DG, are essential for the analysis of antenna elements in a MIMO system. These parameters collectively provide a validation of system performance and efficiency. The ECC calculates the correlation between closely spaced antenna elements in a MIMO system. Although zero correlation is theoretically desired, this condition is not practically attainable; therefore, ECC values below 0.5 are generally considered acceptable. ECC can be calculated from either S-parameters or radiation patterns. While S-parameter-based calculations are widely used, they are valid only for lossless antennas in an isotropic environment. Printed antennas, particularly those operating inside the human body, exhibit significant losses, which can lead to inaccurate ECC calculation when relying on S-parameters. In contrast, the radiation-pattern-based method provides a more accurate representation because it reflects the true channel characteristics, considering antenna losses, spatial behavior, and mutual coupling effects. It also maintains the actual radiative properties and their influence on channel performance, making it a more reliable evaluation method. In this work, the ECC is determined using the far-field radiation patterns of the proposed antenna using the following equation:

(4) $$ECC=\frac{{\left|\iint_{0}^{4\phi }\left[{\vec{A}}_{xi}\left(\theta ,\phi \right)\times {\vec{A}}_{yj}\left(\theta ,\phi \right),d\Omega \right]\right|}^{2}}{\iint_{0}^{4\phi }{\left|{\vec{A}}_{xi}\left(\theta ,\phi \right)\right|}^{2},d\Omega \iint_{0}^{4\phi }{\left|{\vec{A}}_{yj}\left(\theta ,\phi \right)\right|}^{2},d\Omega }$$

where ${\vec{A}}_{xi}$ and ${\vec{A}}_{yj}$ are the 3-D radiation patterns. As shown in Figure 10c, ECC values are lower than the standard 0.5 across all bands. These low ECC values indicate excellent isolation within the proposed MIMO system, confirming its suitability for implantable medical devices that require reliable, high-data-rate communication. The DG is another significant parameter that calculates the improvements in signal-to-interference ratio achieved through diversity techniques, ensuring rigorous antenna performance without losses. A DG value of 10 dB is generally considered optimal, corresponding to a correlation between antenna elements. The DG can be computed using the following equation:

(5) $$DG=10\sqrt{1-{ECC}^{2}}$$

As shown in Figure 10d, the proposed MIMO antenna DG values exceed 9 dB, demonstrating good diversity performance and confirming the antenna’s suitability for robust implantable communication.

## 8. Conclusions

This work presents a compact triple-band four-port MIMO antenna for implantable biomedical devices, including WCE and leadless pacemakers. The design addresses the challenges of miniaturization, multiband operation, and inter-element isolation without employing any external component within the constrained IMBD environment. Operating in the WMTS and ISM bands, the proposed MIMO antenna achieved good performance and supports high-data-rate telemetry. SAR analysis confirms compliance with human-body safety limits. Extensive full-wave simulations were performed in both homogeneous and heterogeneous body phantoms, including fully encapsulated implants. These simulations encompass the design and optimization of the single antenna element, its conversion to a two- and four-element MIMO configuration, and decoupling mechanisms. Wireless communication and MIMO channel analysis, including ECC and DG, demonstrate the system’s ability to support reliable communication exceeding 20 m with data rates up to 100 Mbps. The combination of compactness, triple-band operation, low coupling, and good diversity performance positions the proposed MIMO antenna as a promising solution for next-generation implantable biomedical devices, offering both high efficiency and safety for real-time diagnostic and therapeutic applications. The study on measured S-parameters, radiation pattern, and received power will be carried out in future work.

## Figures and Tables

**Table 1:** Comparison with reported MIMO implantable antennas.

Ref.	Size [mm^3^]	Freq. [GHz]	ISO. [dB]	Gain [dB]	Depth [mm]	Tissue
[13]	307	0.403	−26	−36	4	1-layer
[14]	441.27	2.45	−16.9	−15.2	19.5	3-layer
[15]	120.58	1.4, 2.45	−23, −24	−28.17	50	Heart
				−18.15		GI
[16]	280.035	0.433	−37	−21.8	3	1-layer
[17]	3375	2.45, 5.8	−37, −32	−18.5	50	3-layer
[18]	301.716	1.47	>−18.15	−27.09	25	GI
		2.45	>−21.1	−29.73		
[23]	28.1	0.915	>35.85	−29.8	50	GI
		2.45	>31.6	−24.6		
[24]	14.7	0.915	>35	−30.47	17	Brain
		2.45	>27	−24.71		
		1.4	−37.74	−35		
This work	18.785	2.45	−44.54	−25	50	Heart, GI
		5.8	−26.48	−15		

## Data Availability

The data are available upon reasonable request.
